# Supplementary material for: Splice-Junction-Based Mapping of Alternative Isoforms in the Human Proteome
Source: Cell Rep. Author manuscript; Available in PMC 2020 Jan 15. (PMC6961840; doi:10.1016/j.celrep.2019.11.026)
Supplement: 2 [file NIHMS1546469-supplement-2.zip › DF1/PXD006675/RightVentricle/RightVentricle_4_AAK1_TAAEDSNLISGFDVPEGSDK.pdf]

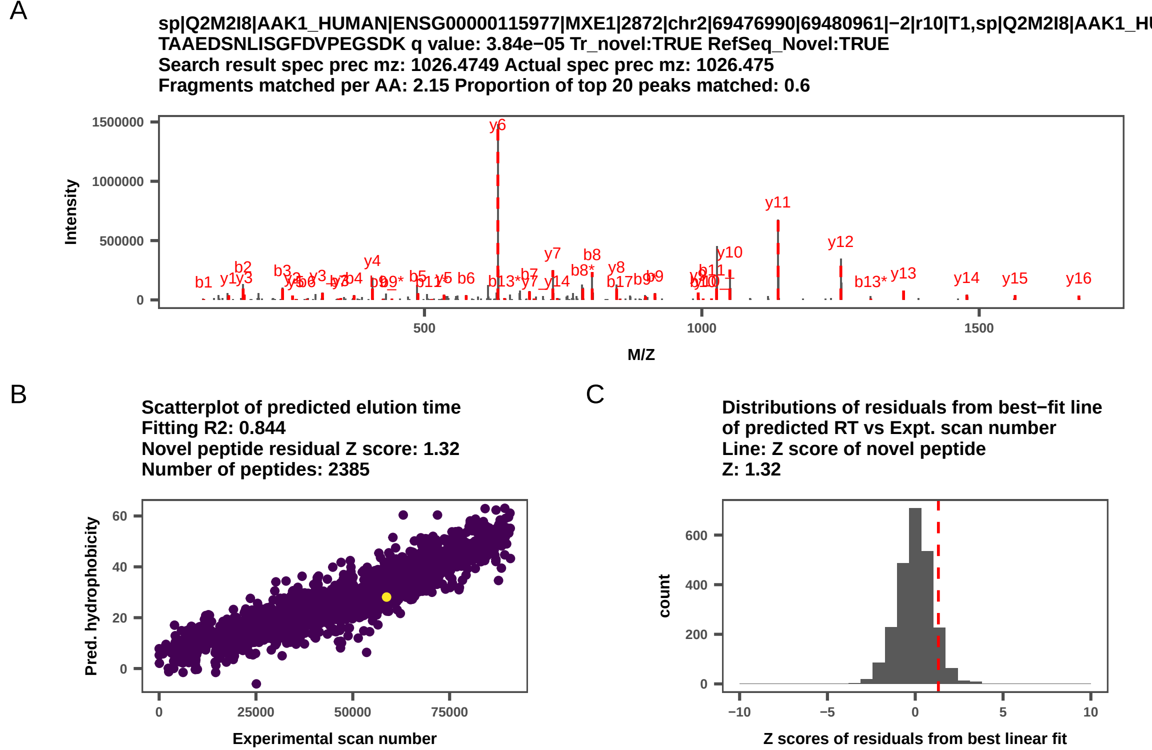
A
sp|Q2M2I8|AAK1_HUMAN|ENSG00000115977|MXE1|2872|chr2|69476990|69480961|−2|r10|T1,sp|Q2M2I8|AAK1_HU
TAAEDSNLISGFDVPEGSDK q value: 3.84e−05 Tr_novel:TRUE RefSeq_Novel:TRUE
Search result spec prec mz: 1026.4749 Actual spec prec mz: 1026.475
Fragments matched per AA: 2.15 Proportion of top 20 peaks matched: 0.6
Intensity
1500000
1000000
500000
0
500
1000
1500
M/Z
y6
y11
y12
y10
y7
b8
y13
y14
y15
y16
B
Scatterplot of predicted elution time
Fitting R2: 0.844
Novel peptide residual Z score: 1.32
Number of peptides: 2385
Pred. hydrophobicity
60
40
20
0
0
25000
50000
75000
Experimental scan number
C
Distributions of residuals from best−fit line
of predicted RT vs Expt. scan number
Line: Z score of novel peptide
Z: 1.32
count
600
400
200
0
−10
−5
0
5
10
Z scores of residuals from best linear fit
